# A Cross-Sectional Study of Companion Animal-Derived Multidrug-Resistant Escherichia coli in Hangzhou, China

**DOI:** 10.1128/spectrum.02113-22

**Published:** 2023-02-22

**Authors:** Lin Teng, Mengyao Feng, Sihao Liao, Zhijie Zheng, Chenghao Jia, Xin Zhou, Reshma B. Nambiar, Zhengxin Ma, Min Yue

**Affiliations:** a Department of Veterinary Medicine, Zhejiang University College of Animal Sciences, Hangzhou, China; b Mount Desert Island Biological Laboratory, Bar Harbor, Maine, USA; c State Key Laboratory for Diagnosis and Treatment of Infectious Diseases, National Clinical Research Center for Infectious Diseases, National Medical Center for Infectious Diseases, The First Affiliated Hospital, College of Medicine, Zhejiang University, Hangzhou, China; d Hainan Institute of Zhejiang University, Sanya, China; e Zhejiang Provincial Key Laboratory of Preventive Veterinary Medicine, Hangzhou, China; BGI Group

**Keywords:** *Escherichia coli*, multidrug resistance, carbapenem resistance, multilocus sequence type, companion animals, China

## Abstract

Antimicrobial resistance poses a challenge to global public health, and companion animals could serve as the reservoir for antimicrobial-resistant bacteria. However, the prevalence of antimicrobial-resistant bacteria, especially multidrug-resistant (MDR) bacteria, and the associated risk factors from companion animals are partially understood. Here, we aim to investigate the prevalence of MDR Escherichia coli, as an indicator bacterium, in pet cats and dogs in Hangzhou, China, and evaluate the factors affecting the prevalence of MDR E. coli. The proportion of pets carrying MDR E. coli was 35.77% (49/137), i.e., 40.96% (34/83) for dogs and 27.28% (15/54) for cats. Isolates resistant to trimethoprim-sulfamethoxazole (49.40% and 44.44%), amoxicillin-clavulanic acid (42.17% and 38.89%), and nalidixic acid (40.96% and 35.19%) were the most prevalent in dogs and cats. Interestingly, comparable prevalence of MDR E. coli was observed in pet dogs and cats regardless of the health condition and the history of antibiotic use. Genetic diversity analysis indicates a total of 86 sequencing types (23 clonal complexes), with ST12 being the most dominant. Further genomic investigation of a carbapenem-resistant E. coli ST410 isolate reveals abundant antimicrobial-resistance genes and a plasmid-borne carbapenemase gene (NDM-5) flanked by insertion sequences of IS*91* and IS*31*, suggesting the plasmid and insertion sequences may be involved in carbapenem-resistance dissemination. These data show that companion animal-derived MDR bacteria could threaten public health, and further regulation and supervision of antimicrobial use in pet clinics should be established in China.

**IMPORTANCE** MDR Escherichia coli are considered a global threat because of the decreasing options for antimicrobial therapy. Companion animals could be a reservoir of MDR E. coli, and the numbers of pets and households owning pets in China are booming. However, the prevalence and risk factors of MDR E. coli carriage in Chinese pets were rarely studied. Here, we investigated the prevalence of MDR E. coli in pets in Hangzhou, one of the leading cities with the most established pet market in China, and explored the factors that affected the prevalence. Our findings showed high prevalences of MDR E. coli in pet dogs and cats regardless of their health condition and the history of antibiotic use, suggesting their potential role of public health risk. A call-to-action for improved regulation of antimicrobial use in companion animal is needed in China.

## INTRODUCTION

Antimicrobial resistance is one of the most significant global challenges, resulting in at least 700,000 death per year (1–3). Antimicrobial-resistant Escherichia coli is one of the leading problematic bacteria worldwide (4). Based on the data from the Chinese Antimicrobial Surveillance Network (CHINET) in 2021, 57,245 antimicrobial-resistant E. coli isolates were recovered from clinical settings, consisting of 18.96% of overall clinical antimicrobial-resistant isolates identified (5). Currently, antimicrobial-resistant E. coli remains an immense disease burden in China (6). The emergence and dissemination of these bacteria were partially promoted by the misuse and overuse of antimicrobials in humans, food animals, and pets (7). Therefore, the One Health approach or efforts are urgently needed to address such a problem, and much knowledge regarding companion animals is largely lacking (8, 9).

Companion animals could be a reservoir of antimicrobial-resistant bacteria and serve as vehicles for further dissemination to humans (10). They carry a variety of antibiotic-resistant pathogens, including extended-spectrum beta-lactamase (ESBL)-producing E. coli, carbapenem-resistant E. coli, methicillin-resistant Staphylococcus aureus, and ESBL-producing Klebsiella pneumoniae (11–15). These pet-derived bacteria belong to antimicrobial-resistant “priority pathogens” that pose significant threats to human health (16). In addition, companion animals, especially dogs and cats, directly contact each other and their owners at a high frequency, leading to an increased chance of zoonotic transmission of antimicrobial-resistant bacteria among themselves and their owners (17). Based on the One Health concept, to prevent humans from pet-derived antimicrobial-resistant bacterial infections, it is critical to understand the distribution and antimicrobial resistance profiles of antimicrobial-resistant bacteria in companion animals, and identify the associated risk factors contributing to the development of antimicrobial-resistant bacteria, and consequently establish the control measures. The presence and resistance profile of the MDR bacteria in companion animals were studied worldwide, including in Europe, the United States, Brazil, and Thailand (18–21). However, the genetic diversity of the MDR bacteria population, especially the carbapenem-resistant E. coli, of pet origins in China was rarely examined (22).

Misuse and overuse of antimicrobials could be the primary reasons driving the high prevalence of pet-derived MDR bacteria in companion animals (23, 24). Even though regulations for the use of antibiotics in pets is available, antibiotic misuse or overuse remains a key issue in veterinary clinics. Notably, the antibiotics used for human medicine are also frequently used for pets (25). Therefore, the first-line antimicrobials for infection treatment in humans may be distributed to pets. Currently, few studies have investigated companion animals in China to understand the parameters of MDR bacteria development, including the association between MDR bacteria prevalence and antimicrobial therapy and health conditions.

In the current study, we investigated a pet hospital in Hangzhou, China, to understand the prevalence, phenotypic resistance, and genetic diversity of MDR E. coli in pets with distinct health conditions and a history of antimicrobial therapies. Furthermore, whole-genome sequencing (WGS) and genomic investigation were conducted to explore the contribution of antimicrobial-resistance genes and mobile genetic elements to the presence of carbapenem-resistant bacteria in pets.

## RESULTS

### Prevalence of multidrug-resistant E. coli.

We identified E. coli isolates from 78 out of 137 pet dogs (*n* = 83) and cats (*n* = 54). A total of 276 E. coli isolates were recovered from fecal samples of dogs and cats of versatile breeds, ages, and health conditions (Table S1). More than one isolate was collected from an animal if it visited the pet hospital more than once (i.e., one representative isolate each time, Data set 1). Out of the 276 isolates, 113 (40.94%) were recovered from cats, and 163 (59.06%) were collected from dogs (Table S1). Besides, the antimicrobial treatment history in the previous month was investigated, with 25 animals treated with antimicrobials and 112 animals receiving no antimicrobial treatment.

**FIG 1:**
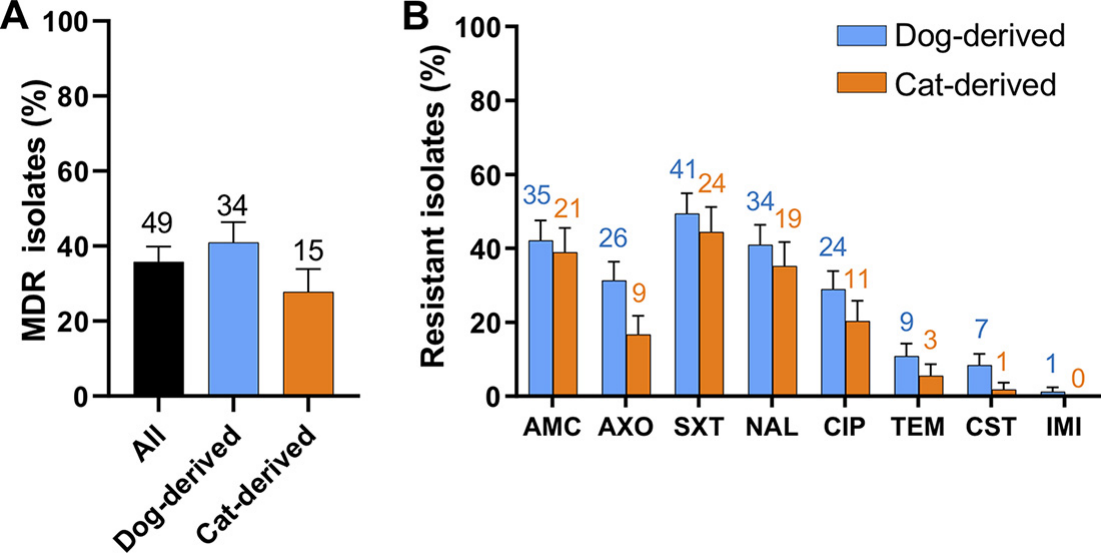
The percentage of antimicrobial-resistant Escherichia coli from pets. (A) The percentage of pets carrying multidrug-resistant E. coli. (B) The percentage of E. coli resistant to each tested antibiotic. Data are presented as mean ± SE. The two-tailed *P*-values were calculated by the Chi-squared test. The number above each bar is the exact number of positive samples that contain MDR isolates or antimicrobial-resistant isolates.

To investigate whether these isolates were antimicrobial-resistant, we conducted antimicrobial susceptibility testing using eight available antimicrobials, including amoxicillin-clavulanic acid (AMC), ceftriaxone (AXO), trimethoprim-sulfamethoxazole (SXT), nalidixic acid (NAL), ciprofloxacin (CIP), temocillin (TEM), colistin (CST), and imipenem (IMI). All 276 isolates contained 40 antimicrobial resistance profiles (Table S2), among which 251 (90.94%) isolates were resistant to at least one antimicrobial and 137 (49.64%) isolates were resistant to ≥3 classes of antimicrobials, indicating they are MDR (26). To calculate the prevalence of MDR E. coli among dogs and cats, an isolate with the broadest antimicrobial resistance profile was selected from each animal as a representative. In total, 35.77% (49/137, 95% confidence interval [CI] = 0.276 to 0.440) of the pets carried MDR E. coli, i.e., 40.96% (34/83, 95% CI = 0.302 to 0.518) for dogs and 27.78% (15/54, 95% CI = 0.156 to 0.400) for cats (Fig. 1A). Notably, 10 isolates were resistant to ≥6 classes of antimicrobials. We further investigated the percentage of dogs and cats that carry isolates resistant to each antimicrobial. The top three resistance rates were linked to SXT- (dog: 41/83, 49.40%, 95% CI = 0.384 to 0.604; cat: 24/54, 44.44%, 95% CI = 0.309 to 0.580), AMC- (dog: 35/83, 42.17%, 95% CI = 0.313 to 0.530; cat: 21/54, 38.89%, 95% CI = 0.256 to 0.522), and NAL- (dog: 34/83, 49.40%, 95% CI = 0.384 to 0.604; cat: 19/54, 44.44%, 95% CI = 0.309 to 0.580) resistance (Fig. 1B). On the contrary, low resistance rates were observed against TEM (dog: 9/83, 10.84%, 95% CI = 0.040 to 0.177; cat: 23/54, 5.56%, 95% CI = 0 to 0.118), CST (dog: 7/83, 8.43%, 95% CI = 0.023 to 0.145; cat: 1/54, 1.85%, 95% CI = 0 to 0.055), and IMI (dog: 1/83, 1.20%, 95% CI = 0 to 0.036; cat: 0/54, 0%). Collectively, the majority of these isolates were antimicrobial-resistant as well as multidrug-resistant.

### Diseased pets and healthy pets contained comparable antibiotic-resistant E. coli strains.

**FIG 2:**
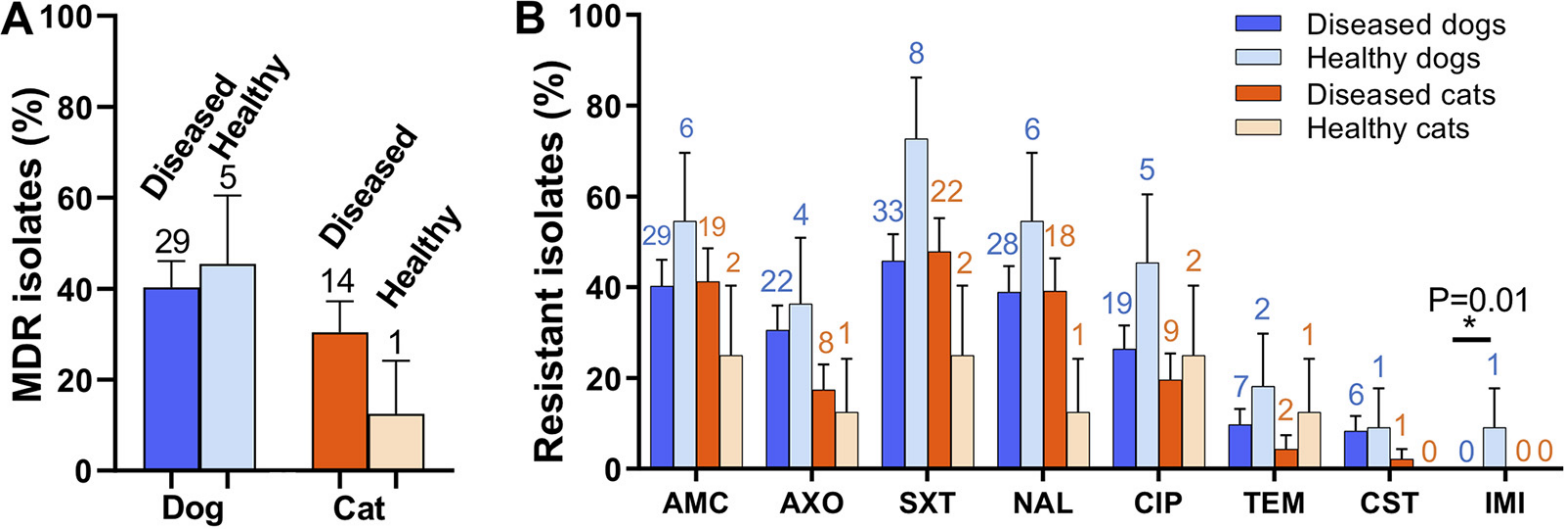
The association between the prevalence of antimicrobial-resistant E. coli and the health condition of pets. (A) The percentage of multidrug-resistant E. coli in pets with distinct health conditions. (B) Resistance rates of isolates from dogs and cats with different health conditions. Data are presented as mean ± SE. The two-tailed *P*-values were calculated using the Chi-squared test. The asterisk represents statistical significance (*P* < 0.05). The number above each bar indicates the exact number of positive samples that contain MDR isolates or antimicrobial-resistant isolates.

To investigate the association between the presence of multidrug-resistant E. coli and animal health conditions, we compared the percentage of MDR E. coli in healthy and sick companion animals. Compared with healthy pets, the diseased animals showed a comparable rate of MDR isolates (Fig. 2A). The percentage of MDR isolates of diseased dogs (40.28%, 29/72, 95% CI = 0.287 to 0.518) was similar to that of healthy dogs (45.45%, 5/11, 95% CI = 0.154 to 0.755). The percentage of antibiotic-resistance E. coli against each antibiotic, except for IMI, was similar between diseased and healthy dogs (Fig. 2B). Similarly, the percentage of MDR E. coli from diseased cats was 30.43% (14/46, 95% CI = 0.169 to 0.440), while this number was as low as 12.50% (1/8, 95% CI = 0 to 0.359) in healthy cats. Compared with the healthy cat, diseased cats carry comparable percentages of AMC- (41.30%, 95% CI = 0.268 to 0.558 in diseased cats versus 25.00%, 95% CI = 0 to 0.556 in healthy cats), AXO- (17.39%, 95% CI = 0.062 to 0.286 versus 12.50%, 95% CI = 0 to 0.359), SXT- (47.83%, 95% CI = 0.331 to 0.626 versus 25.00%, 95% CI = 0 to 0.556), and NAL- (39.13%, 95% CI = 0.247 to 0.535 versus 12.50%, 95% CI = 0 to 0.557) resistant isolates (Fig. 2B). Taken together, diseased and healthy pets contain comparable antibiotic-resistant isolates.

### AMR E. coli isolates were prevalent in pets with and without recent histories of antimicrobial therapy.

**FIG 3:**
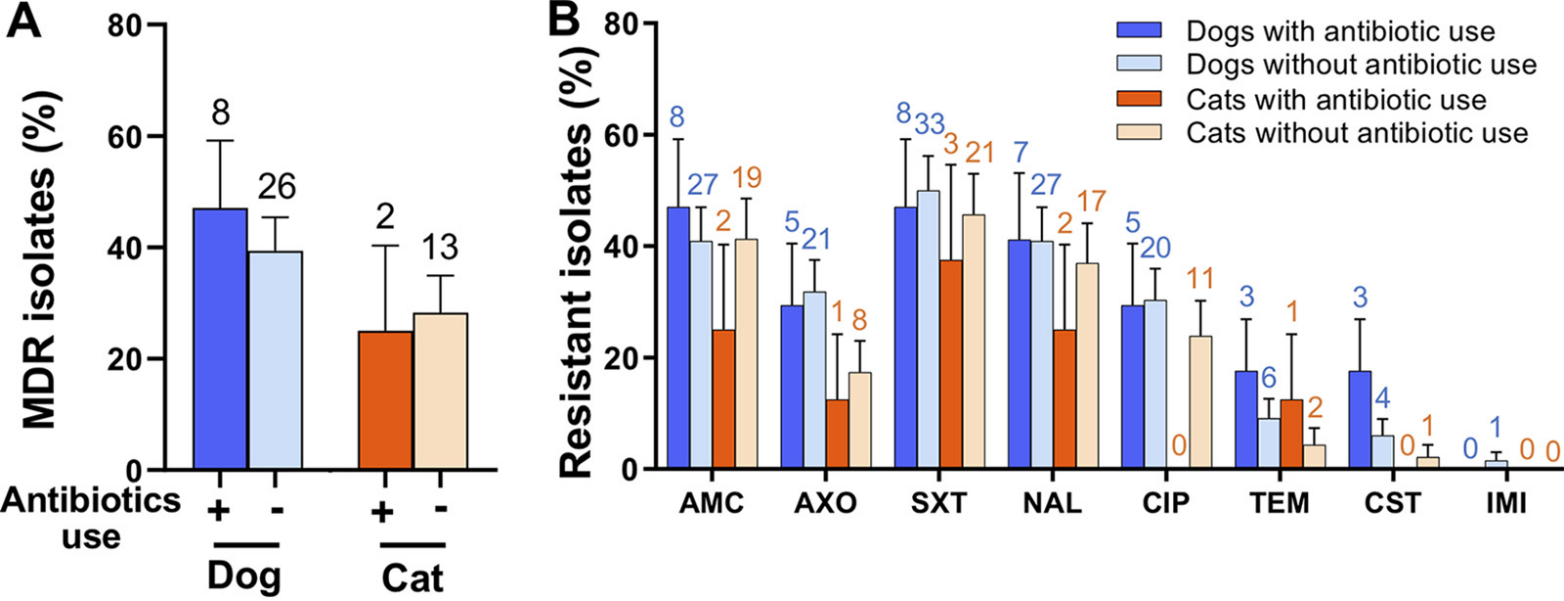
Association between the prevalence of antimicrobial-resistant E. coli and antimicrobial treatment history. (A) Percentage of multidrug-resistant E. coli in pets with an antimicrobial treatment history within a month. (B) Resistance rates of isolates from dogs and cats with an antimicrobial treatment history within a month. Data are presented as mean ± SE. The *P*-values were calculated using the Chi-squared test. The number above each bar represents the exact number of positive samples that contain MDR isolates or antimicrobial-resistant isolates.

Antimicrobial therapies for animals can select antimicrobial-resistant bacteria (27). To investigate the effect of antimicrobial treatment on the presence of antimicrobial-resistant bacteria in pets, we categorized the pets into two groups depending on whether they received the antimicrobial treatment 1 month before visiting the animal hospital. No significant difference was observed in the percentage of MDR isolates between pets with and without antimicrobial treatment (*P* > 0.05). The dogs treated with antimicrobials carried a similar (*P* > 0.05) percentile of MDR isolates (47.06%, 8/17, 95% CI = 0.228 to 0.713) with dogs that received no antimicrobial treatment in the previous month (39.39%, 26/66, 95% CI = 0.274 to 0.514). Compared with the isolates in the dogs that received no antimicrobial, the E. coli in the dogs with antimicrobial treatment contained comparable percentages of antimicrobial resistance against AMC (47.06%, 95% CI = 0.228 to 0.713 in dogs with antimicrobial use versus 40.91%, 95% CI = 0.288 to 0.530 in dogs with no antimicrobial use), NAL (41.18%, 95% CI = 0.173 to 0.650 versus 40.91%, 95% CI = 0.288 to 0.530), TEM (17.65%, 95% CI = 0 to 0.361 versus 9.09%, 95% CI = 0.020 to 0.162), and CST (17.65%, 95% CI = 0 to 0.361 versus 6.06%, 95% CI = 0.002 to 0.119) (Fig. 3B). Interestingly, the cats treated with antibiotics showed a slightly lower rate (*P* > 0.05) of MDR isolate (25.00%, 2/8, 95% CI = 0 to 0.556) than those received no antibiotics (28.26%, 8/17, 95% CI = 0.150 to 0.415). Except for the TEM and IMI groups, the reduced percentages of resistant isolates were observed in cats with antibiotic treatment (Fig. 3B). Of note, the top three resistance rates in dogs and cats were against STX, AMC, and NAL, plausibly because these are first-line antibiotics that were frequently used in animal hospitals. The lack of substantial difference between animals with and without antibiotic treatment may be because of relatively small sampling size.

### Population diversity of E. coli from pets.

**FIG 4:**
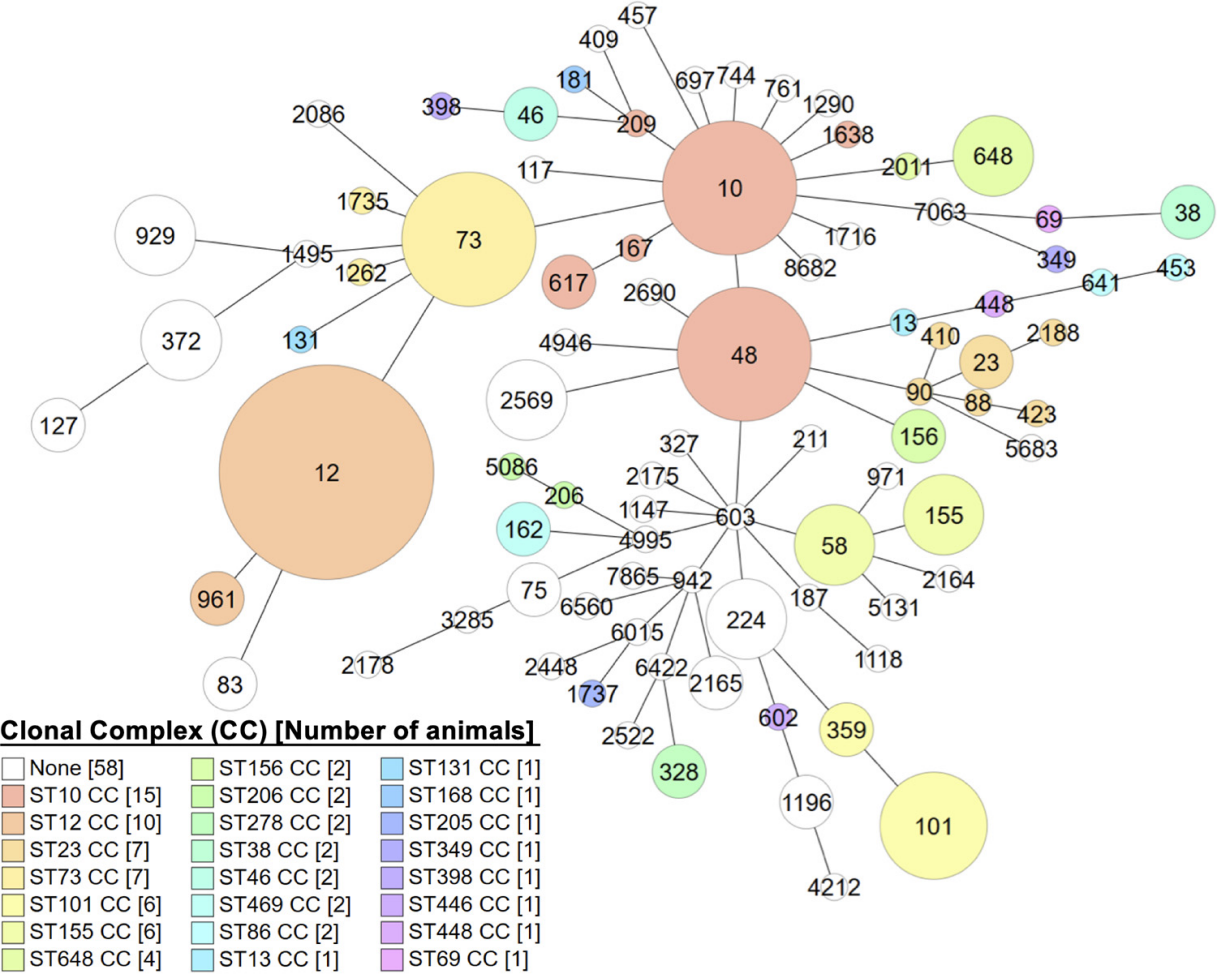
Population diversity of pet-derived E. coli. Each node represents ST, with the sizes of the node in proportion to the number of isolates. Circles in the same colors represent the same ST clonal complex (CC), while circles with distinct colors indicate 23 ST clonal complex (CC).

Sequencing types (ST) of E. coli can be used to reflect bacterial population structure by comparing the genetic sequences of seven housekeeping genes (28, 29). To understand the population diversity of these E. coli isolates, we identified their STs by multilocus sequence typing (MLST). Among all 276 E. coli isolates from 137 animals, 86 STs were identified, including three predominant STs (ST from more than five animals), i.e., ST12 (5.84%, 8/137), ST10 (3.65%, 5/137), and ST73 (3.65%, 5/137). Besides, isolates from eight animals cannot be assigned to any previously known STs, suggesting the presence of novel STs. Additionally, a clonal complex (CC) consists of multiple phylogenetically related STs (29). Except for 45 STs that cannot be assigned to any CC, 41 STs were classified into 23 CCs, indicating the diversity of E. coli isolates from pet dogs and cats. Among the 23 CCs, the top three most prevalent CCs were ST10 CC (9.49%, 13/137, i.e., ST10, ST48, ST167, ST617, ST209, and ST1638), ST12 CC (7.30%, 10/137, i.e., ST12 and ST961), and ST101 CC (4.38%, 6/137, i.e., ST101 and ST359) (Fig. 4; Table S3).

### Host-specific STs and distinct antimicrobial resistance profile.

**FIG 5:**
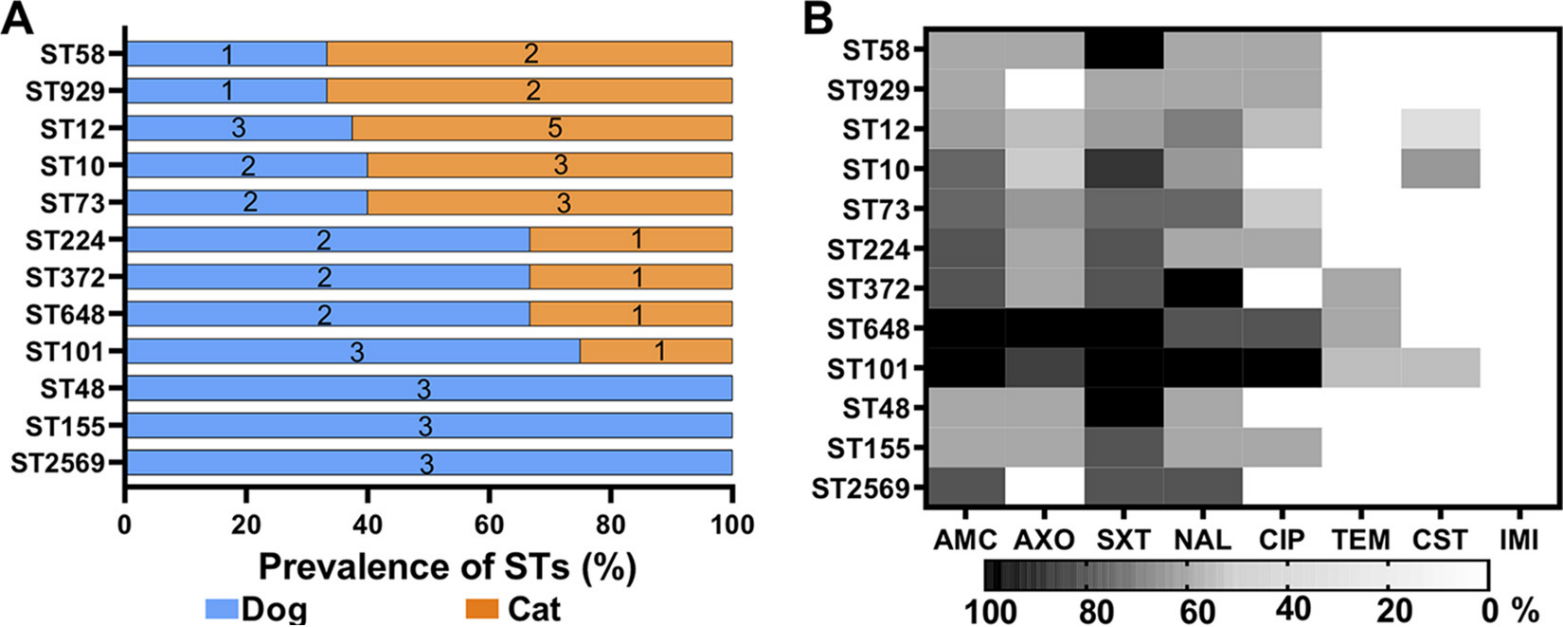
Host-specific sequencing types and antimicrobial-resistance profiles. (A) Distribution of E. coli sequencing types in cats and dogs. (B) Percentage of isolates in each sequencing type resistant to antibiotics. The heatmap shows the percentage of isolates in each ST that is resistant to a panel of antibiotics, including AMC (amoxicillin-clavulanic acid), AXO (ceftriaxone), SXT (trimethoprim-sulfamethoxazole), NAL (nalidixic acid), CIP (ciprofloxacin), TEM (temocillin), CST (colistin), and IMI (imipenem). The number on each bar represents the exact number of positive samples.

We further investigated the host-specific ST by linking the host types with the STs. ST48, ST155, and ST2569 were exclusively identified in dogs (Fig. 5A). Three STs (i.e., ST224, ST372, ST648, and ST101) were more frequently (>60%) identified in the dogs, while the other five STs (ST58, ST929, ST12, ST10, and ST73) were more frequently detected in the cats. Although significant differences were not detected between hosts, which may result from the low sample size, a tendency of host-specific STs was observed. Besides, bacterial STs with a broad spectrum of antimicrobial resistance may pose great threats to their owners and public health. Therefore, we evaluated the antimicrobial-resistant profile of each ST (Fig. 5B). The ST101 (>75.0% isolates) showed the broadest antimicrobial-resistant spectra against AMC, AXO, SXT, NAL, and CIP. SXT resistance was observed in all or a majority of ST58 (100.0%), ST73 (60.0%), ST10 (80.0%), ST648 (100.0%), ST372 (66.7%), ST224 (66.7%), ST101 (100.0%), ST155 (66.7%), ST2569 (66.7%), and ST48 (100.0%) isolates. Notably, 40.0% of ST10 isolates were resistant to CST, used for last-resort antimicrobial therapy against MDR Gram-negative pathogens.

### Genetic characterization of a carbapenem-resistant E. coli.

An isolate from ST410 (20181028-1-12) showed resistance to imipenem, which belongs to the last-resort antimicrobials (carbapenems) to treat infections caused by clinical extend-spectrum beta-lactamase-producing bacteria (30). To investigate genetic factors leading to carbapenem resistance, we conducted WGS for E. coli ST410 isolate 20181028-1-12 and identified its plasmid replicon types and ARGs. This isolate carried a variety of incompatibility (Inc) plasmid types, including IncFIA, IncFIB, IncFII, IncI1, and IncX1. Besides, we identified ARGs conferring bacterial resistance to aminoglycoside, beta-lactam, phenicol, trimethoprim, macrolide, quinolone, sulfonamide, and tetracycline (Table 1). A carbapenemase-producing gene *bla*_NDM-5_ was detected and flanked by the IS*91* transposase gene (*tnpA*_IS*91*) and the IS*30* transposase gene (*tnpA*_IS*30*) in its plasmid DNA (Fig. S1; Data set 2), indicating the mobile genetic elements, i.e., insertion sequences and plasmids, may drive the acquisition of *bla*_NDM-5_ in this pet-derived isolate.

**TABLE 1:** Antimicrobial-resistant genes carried by carbapenem-resistant E. coli isolate 20181028-1-12

Gene type	Gene function	Gene name
Antimicrobial-resistant genes (*n* = 22)	Aminoglycoside resistance	*aac(3)-Iid, aadA2, aph(3″)-Ib, aph(6)-Id, aadA5* ^*a*^
	Beta-lactam resistance	*bla*CMY-2, *bla*CTX-M-15, *bla*NDM-5, *bla*OXA-1, *bla*TEM-1B
	Phenicol resistance	*FloR*
	Trimethoprim resistance	*dfrA17, dfrA12* ^*a*^
	Macrolide resistance	*mdf(A), mph(A)*
	Quinolone resistance	*oqxA, oqxB*
	Sulphonamide resistance	*sul1, sul2, sul3*
	Tetracycline resistance	*tet(A)* ^*a*^ *, tet(B)* ^*a*^

a AMR gene found on the plasmid sequences.

## DISCUSSION

In this study, we investigated a pet hospital in Hangzhou, China, in 2018 and obtained 276 E. coli isolates from the fecal samples of pet cats and dogs. The prevalence of cats and dogs that carried MDR E. coli were 27.78% (95% CI = 0.156 to 0.400) and 40.96% (95% CI = 0.302 to 0.518), respectively. We observed high prevalences of MDR E. coli in diseased and healthy pet dogs and cats, indicating they are a reservoir for antimicrobial-resistant bacteria. Besides, we grouped the pets (i.e., 54 cats and 83 dogs) depending on whether they received antimicrobial treatment 1 month before they visited the pet hospital, showing that similar percentages of MDR E. coli were identified in the pets regardless of antimicrobial treatment history (i.e., 25.00%, 95% CI = 0 to 0.556 versus 28.26%, 95% CI = 0.150 to 0.415 for cats with and without antimicrobial treatment, respectively; 47.06%, 95% CI = 0.228 to 0.713 versus 39.39%, 95% CI = 0.274 to 0.514 for dogs with and without antimicrobial treatment, respectively). By MLST, all isolates were identified as 86 STs, among which 41 STs belong to 23 clonal complexes. The ST48, ST155, and ST2569 isolates were only identified from dogs, suggesting a potential host specificity of these isolates. Notably, the ST101 isolates showed resistance to a broad range of antimicrobials, indicating their threats to public health.

Pets represent a reservoir of MDR bacteria, and a high prevalence of antimicrobial-resistant E. coli was identified in companion animals in different countries (22, 31–34). In this study, 27.78% (95% CI = 0.156 to 0.400) of cats and 40.96% (95% CI = 0.302 to 0.518) of dogs in Hangzhou, China, contained MDR E. coli (Fig. 1A). Interestingly, Chen et al. investigated 1,886 samples from diseased pets in Beijing, China, and identified 79 (4.19%) MDR E. coli isolates from 2012 to 2017. In Korea, a study that collected 877 intestinal samples of dogs found MDR isolates from 32% and 48% of stray dog-derived and hospitalized pet dog-derived E. coli, respectively. Saputra et al. identified clinical isolates from 22 veterinary diagnostic laboratories in Australia and reported that 18.1% of dog-derived E. coli and 11.7% of cat-derived E. coli were MDR (32). The ratio of MDR E. coli from pets in Poland and the United States was reported as 66.8% and 52.0%, respectively (33, 34). As different antimicrobial panels were used to detect MDR E. coli, the percentages of MDR E. coli are not comparable among studies. However, MDR E. coli from pets was generally high, suggesting these bacteria may threaten public health.

E. coli with a broad range of antimicrobial resistance were detected in the pets. In the current study, 35.77% of representative E. coli isolates were resistant to more than three classes of antimicrobials (Fig. 1A), and 12.82% of the isolates were resistant to more than six classes of antimicrobials (Table S2), indicating limited drugs were available for antimicrobial treatment for the pets infected by these MDR isolates. A large proportion of isolates were resistant to the first-line antimicrobials in veterinary medicine (e.g., trimethoprim-sulfamethoxazole and nalidixic acid), and isolates were resistant to last-resort antimicrobials (i.e., colistin) were also identified. Similarly, 8.7% (49/566) of colistin-resistant *Enterobacteriaceae* were identified from 1,439 nasal and rectal swab samples of dogs and cats in Beijing, China (35). A recent study collected rectal samples from dogs and their owners, finding that 2.7% of the samples contained colistin-resistant E. coli (4, 35, 36). These pet-derived colistin-resistant bacteria may emerge from the misuse of colistin in pet clinics or pet foods, as colistin was used for decades in animal feed (37). Besides, an association between a high prevalence of MDR E. coli and pets’ health condition (or antimicrobial treatment history) was not observed (Fig. 2; Fig. 3), which might be because of the small sample size. A study in Finland found that staphylococcal isolates from dogs with antimicrobial treatment were more resistant to trimethoprim-sulfamethoxazole than the isolates from dogs with no antimicrobial treatment (38). A longitude study reported an immediate and significant increase of third-generation cephalosporin resistance, AmpC-producing, MDR- and/or fluoroquinolone-resistant E. coli in the feces of dogs after they were treated with β-lactams or fluoroquinolones (27). These results emphasize that the use of antimicrobials in pet clinics should be well-supervised to avoid the increase of pet-derived MDR-resistant bacteria.

The presence of carbapenem-resistant E. coli was detected in a companion animal in our current study (Table 1), as well as the studies in the United States and Germany (14, 15). Multiple factors may play roles in the presence of this bacterium in pets. Although carbapenems are not registered antimicrobials for animal use (39), the misuse of these antimicrobials in pets may result in the presence of these bacteria. Besides, hospitalized companion animals can serve as a source of these carbapenem-resistant bacteria (40). Even though environmental samples were not collected in this study, the hospital environments contaminated by this bacterium might promote bacterial transmission among individual pets (40).

Although fecal samples of pet owners were not collected in this study, the transmission of MDR E. coli among pets and their owners was observed in multiple studies (17, 36, 41). The colistin-resistant E. coli with the same pulsed-field gel electrophoresis pattern were identified from an employee, dogs, and cats in the same pet store, suggesting the bacterium transferred between pets and humans (17). Similarly, a study in Finland reported identical carbapenemase-producing E. coli clones in pets and humans using WGS and core genome MLST (41). By identifying clonal colistin-resistant E. coli isolates using WGS, Lei et al. proved the cross-species transmission of this bacterium between a dog and its owner (36). Collectively, these results emphasize that the transmission of antimicrobial-resistant bacteria between humans and pets and correlated disease burdens cannot be underestimated.

In the current study, we identified diverse STs of E. coli from pets, including ST10, ST12, ST48, ST58, ST73, ST101, and ST131 (Fig. 4A). ST12 CC, ST10 CC, and ST101 CC were the most predominant among pet-derived isolates in our study. The predominant ST12 CC isolates were identified from a wide range of hosts, including hospitalized humans, birds of prey, and dogs, and have a high chance of carrying diverse ARGs or being MDR (42–44). Notably, the ST12 CC isolates were identified from bloodstream infection cases in the United States (45). They were one of the most common lineages associated with bacteremia of humans in England (42), suggesting the virulence potential of ST12 CC isolates. Similarly, E. coli ST10, ST73, ST101, and ST131 were isolated not only in cats and dogs (22, 36, 46) but also in patients, suggesting their pathogenicity. E. coli ST10 CC isolates were identified from urine samples and blood samples of patients, as well as retail meat and beef cattle (45, 47, 48). Adams-Sapper et al. reported that ST73 CC isolates were detected in humans with bloodstream infections, and seven out of 35 ST73 CC isolates were MDR (45). ST101 CC isolates were identified from patients with urinary tract infections, retail meat, and migratory bird (*Hirundo rustica*) (47, 49). Notably, ST131 is one of the most predominant and widely spread E. coli lineages worldwide and is frequently identified as the causative agent of urinary tract infections (UTIs) in humans (50), suggesting the pet-derived ST131 isolates may be virulent and cause severe disease in humans and animals.

Overall, our study reported a high prevalence of MDR E. coli recovered from healthy and diseased cats and dogs in Hangzhou, China. These pet-derived MDR isolates belong to versatile STs and CCs. The carbapenem-resistant E. coli emerging in companion animals may result from the misuse of antimicrobials. Our data suggest that pet-derived MDR isolates pose public health risks with increasing concerns, and antimicrobials should be regulated at a higher level in small animal clinics.

## MATERIALS AND METHODS

### Ethics statement.

This study was endorsed by the Zhejiang University Animal Ethics Committee with the approval document (ZJU20190094).

### Sample collection and bacterial identification.

This study collected fecal samples of dogs and cats of versatile breeds in one of the biggest pet hospitals, the Veterinary Teaching Hospital of Zhejiang University in Hangzhou, between March and December 2018 (51). As the population proportion of MRD E. coli in dogs and cats were rarely evaluated, few available proportions were available for calculating minimal sample sizes. Therefore, we used the average population proportion of MDR E. coli from dogs and cats (4.2% for both dogs and cats, i.e., 79 dogs and cats carrying MDR E. coli from a total of 1,886 dogs and cats in Beijing, China from 2012 to 2017) reported by Chen et al. (22). The minimal sample sizes for dogs and cats in this study were calculated using the parameters of “confidence level = 95%; margin of error = 5%; power = 0.8, and population proportion = 4.2% (for both dogs and cats)” (https://www.calculator.net/sample-size-calculator.html), resulting in a minimal sample size of 62 for both dogs and cats. We recorded detailed information about these dogs and cats, including their ages, genders, breed, and usage of antimicrobials (Table S4). When the pets visited the hospital, one fecal sample from each animal was collected. For animals visiting the hospital multiple times, one fecal sample was collected during each visit. The feces (*n* = 276) were collected from the rectum of the 137 companion dogs (*n* = 83) and cats (*n* = 54) using two sterile cotton swabs. The swabs were stored in sterile 15-mL conical tubes at 4°C and shipped to the lab within 4 h. To process the samples, 2 mL of sterile phosphate buffered saline (PBS) was added to each conical tube containing cotton swabs with fecal samples. LB broth and buffered peptone water (Oxoid, Hampshire, England) were used for primary culture (37°C for 12 to 16 h) with a dilution ratio of 1:50. The samples were then subcultured on brain heart infusion (BHI) broth at 37°C for 24 h, followed by streaking 100 μL of cell culture on MacConkey Agar at 37°C for 24h. The presumptive E. coli colonies (red and pink colonies on MacConkey Agar) were picked and purified on BHI agar overnight at 37°C. The confirmed pure colonies were mixed with 50% glycerol and stored at −80°C. The suspected E. coli colonies were confirmed using Matrix-assisted laser desorption/ionization time of flight mass spectrometry (MALDI-TOF-MS) biotyper (Bruker, Bremen, Germany). The sample sizes for dogs and cats were 83 and 54, respectively. The power of MDR E. coli for dogs and cats was calculated using an online tool (http://powerandsamplesize.com/Calculators/Test-1-Proportion/1-Sample-Equality), resulting in the power of 0.9980 for dogs (parameters: sample size = 83; type I error rate = 5%; true proportion = 0.278; null hypothesis proportion = 0.042) and 0.9998 for cats (parameters: sample size = 54; type I error rate = 5%; true proportion = 0.41; null hypothesis proportion = 0.042).

### Antimicrobial susceptibility testing.

Isolates from several visits of an animal may be distinct clones which may show different patterns of antimicrobial resistance. Therefore, we conducted antimicrobial susceptibility testing for all 276 isolates to select a representative isolate (an E. coli isolate that showed the broadest antimicrobial resistance profile) from each animal to calculate the prevalence of MDR E. coli among dogs and cats. The phenotypic antimicrobial susceptibility of these E. coli isolates was conducted using the microdilution method according to the Clinical & Laboratory Standards Institute (CLSI) guideline as previously described (52, 53). In the assay, we tested bacterial susceptibility to eight antimicrobials, including amoxicillin/clavulanic acid (AMC, 0.5/0.25 to 64/32 mg/L), ceftriaxone (AXO, 0.015 to 8 mg/L), trimethoprim/sulfamethoxazole (SXT, 0.125/2.375 to 16/304 mg/L), nalidixic acid (NAL, 0.5 to 64 mg/L), ciprofloxacin (CIP, 0.015 to 8 mg/L), temocillin (TEM, 0.06 to 16 mg/L), colistin (CST, 0.06 to 8 mg/L), and imipenem (IMI, 0.015 to 8 mg/L) (Sangon, Shanghai, China). The isolates were determined as resistant, intermediate resistant, and susceptible based on the breakpoints in the guideline of CLSI (54). Isolates resistant to more than three antimicrobial classes were considered multidrug-resistance (26). The MIC assay was also conducted for the control strains E. coli ATCC 25922 and Pseudomonas aeruginosa ATCC 27853, as suggested by CLSI. Isolates resistant to more than three classes of antibiotics were MDR. The animals containing at least one MDR E. coli from over two hospital visits were considered MDR E. coli positive. The prevalence of dog (or cat) carrying MDR E. coli was calculated using the following formula: Prevalence of dog (or cat) carrying MDR E. coli = The number of dogs (or cats) carrying MDR E. coli/total number of dogs (or cats) × 100%.

### Multilocus sequence typing of E. coli strains.

Multilocus sequence typing of E. coli isolates was determined by PCR amplification of seven conserved housekeeping loci (*adk*, *fumC*, *gyrB*, *icd*, *mdh*, *purA*, and *recA*). Bacterial DNA was extracted from overnight culture (BHI broth, 37°C,18h) using a genomic DNA Extraction kit (DP302, TianGen, Beijing, China) according to the manufacturer’s protocol. The PCR procedure was as follows: The 25 μL reaction mixture consisted of 12.5 μL of 2×Power Tap PCR Master Mix (Bioteke Corporation, China), 1 μL of both forward and reverse primers, 1 μL template DNA, 9.5 μL ddH_2_O The PCR was performed under the amplification conditions: predenaturation at 95°C for 2 min, then a serial set of denaturation (95°C for 30 s), annealing (54 to 60°C for 1 min), extension (72°C for 2 min) with 30 cycles, and final extension at 72°C for 5 min. After Sanger sequencing, the forward and reverse sequences were assembled, and the sequences of seven merged housekeeping loci were uploaded to MLST 2.0 in the Center for Genomic Epidemiology (http://www.genomicepidemiology.org/) to identify their sequence types (STs) as previously described (55). All the STs were then submitted to the E. coli MLST database (https://enterobase.warwick.ac.uk/) and categorized into different clonal complexes. Only the nonredundant STs from the same animal were recorded. For instance, if the STs of the second and following isolates were the same as that of the first isolate, only the first isolate was collected to analyze the population structure of E. coli. The phylogenetic relatedness of these STs was analyzed by importing a housekeeping gene matrix of all E. coli isolates to GrapeTree with a selection of MSTree V2 (56).

### Whole-genome sequencing and bioinformatic analyses.

WGS and bioinformatic analyses were conducted as described previously (57–59). One carbapenem-resistant E. coli isolate was chosen for WGS (59–61). The DNA library was constructed and sequenced using an Illumina Hiseq platform to obtain 150 bp pair-end reads (62–64). Quality control of the raw reads was conducted using FastQC (N50 = 155,956 bp), and a contamination screen was done using Kraken2, resulting in 96% of reads being mapped to genomic sequences of Enterobacteriaceae. The raw reads were trimmed using Trimmomatic (Version 0.38) and assembled using SPAdes (Version 3.0). Assembled bacterial genomic sequence was annotated using the NCBI prokaryotic gene annotation pipeline (65–67). To confirm the species of the isolate, taxonomy identification was conducted by submitting the assembled genome sequence to KmerFinder (https://cge.food.dtu.dk/services/KmerFinder/). The sequence type of the carbapenem-resistant E. coli was identified by MLST of the Center in Genomic Epidemiology (https://cge.food.dtu.dk/services/MLST/). The virulence factor genes and antimicrobial-resistance genes were identified using the Virulence Factor Database (VFDB) and ResFinder 4.0, respectively, in Abricate.

### Statistical analyses.

Statistical significance was determined using the Chi-square test in GraphPad Prism. The cutoff of significance was *P* < 0.05.

### Data availability.

The WGS of the pet-derived E. coli strain was deposited in NCBI under the BioProject number PRJNA828008 (SAMN27655685).
