# Supplementary material for: A Cross-Sectional Study of Companion Animal-Derived Multidrug-Resistant Escherichia coli in Hangzhou, China
Source: Microbiol Spectr. 2023 Feb 22;11(2):e02113-22. doi: 10.1128/spectrum.02113-22 (PMC10100847; doi:10.1128/spectrum.02113-22)
Supplement: Supplemental file 4 — Figure S1. Download spectrum.02113-22-s0004.pdf, PDF file, 0.3 MB [file spectrum.02113-22-s0004.pdf]

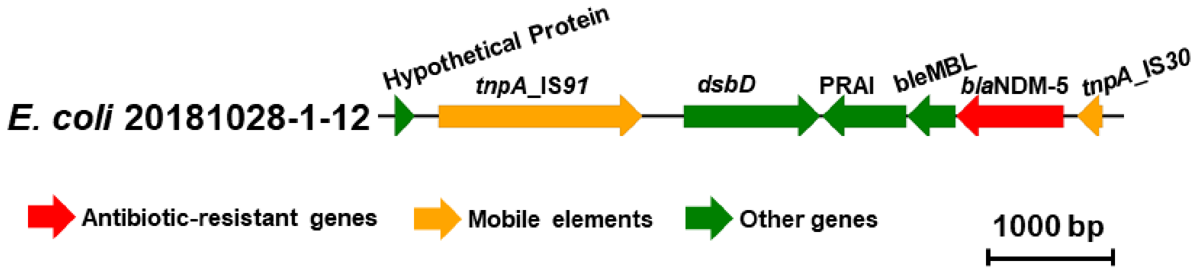

Figure S1. Genetic environments of the *bla*$_{\text{NDM-5}}$ gene in *E. coli* 20181028-1-12.

Table S1. Percentages of *E. coli* in distinct categories of pets.

Category	Sub-category (number of animals)	Number of isolates	Percentage (Isolate No./Total isolate No., %）
Host	Cat (n=54)	113	40.94
	Dog (n=83)	163	59.06
Health condition	Cats with healthy condition (n=8)	16	5.80
	Cats with disease (n=46)	97	35.15
	Dogs with healthy condition (n=11)	23	8.33
	Dogs with disease (n=72)	140	50.72
Use of antimicrobials in the previous month	Yes (n=25)	40	14.49
	No (n=112)	236	85.51

Table S2 Antimicrobial resistance profiles of pet-derived *Escherichia coli* in 2018

Resistance number	Resistance phenotype	Number of isolates	Percentage (%)	Total (%)
0		25	9.06%	9.06%
1	AMC	8	2.90%	17.75%
1	CIP	4	1.45%	
1	CST	1	0.36%	
1	SXT	24	8.70%	
1	NAL	11	3.99%	
1	TEM	1	0.36%	
2	AMC-SXT	24	8.70%	23.55%
2	AMC-NAL	8	2.90%	
2	AMC-AXO	1	0.36%	
2	AXO-SXT	5	1.81%	
2	AXO-NAL	1	0.36%	
2	CIP-SXT	1	0.36%	
2	CST-SXT	1	0.36%	
2	SXT-NAL	17	6.16%	
2	AMC-CST	1	0.36%	
2	AMC-SXT	1	0.36%	
2	CST-SXT	3	1.09%	
2	AMC-CIP-NAL	2	0.72%	
3	AMC-AXO-SXT	8	2.90%	19.20%
3	AMC-AXO-NAL	1	0.36%	
3	AMC-SXT-NAL	15	5.43%	
3	AXO-SXT-NAL	5	1.81%	
3	AMC-CST-SXT	1	0.36%	
3	AMC-SXT-NAL	4	1.45%	
3	AMC-AXO-CIP-NAL	1	0.36%	
3	AMC-CIP-SXT-NAL	13	4.71%	
3	AXO-CIP-SXT-NAL	1	0.36%	
3	CIP-CST-SXT-NAL	4	1.45%	
4	AMC-AXO-SXT-NAL	15	5.43%	18.84%
4	AMC-AXO-NAL-TEM	1	0.36%	
4	AMC-SXT-NAL-TEM	1	0.36%	
4	AMC-CST-SXT-NAL	1	0.36%	
4	AMC-AXO-CIP-SXT-NAL	6	2.17%	
4	AMC-AXO-CIP-SXT-NAL	28	10.14%	

5	AMC-AXO-SXT-NAL-TEM	1	0.36%	10.14%
5	AMC-AXO-CIP-CST-SXT-NAL	1	0.36%	
5	AMC-AXO-CIP-SXT-NAL-TEM	10	3.62%	
5	AMC-AXO-CIP-SXT-NAL-TEM	14	5.07%	
5	AMC-CIP-CST-SXT-NAL-TEM	2	0.72%	
6	AMC-AXO-CIP-CST-SXT-NAL-TEM	4	1.45%	1.45%

Note: The full names of each abbreviation are listed. AMC: Amoxicillin-clavulanic acid, AXO: cefatriaxone, SXT: Trimethoprim-sulfamethoxazole, NAL: Nalidixic acid, CIP: Ciprofloxacin, TEM: Temocillin, CST: Colistin, and IMI: Imipenem

Table S3. Resistance rate of pet-derived *E. coli* to different antimicrobials

Antimicrobial categories	Antimicrobial agents	Abbreviation	Percentage of antimicrobial resistant isolate (Number of isolates)		
			All isolates (n=276)	Dog-derived isolates (n=163)	Cat-derived isolates (n=113)
Penicillins/β-lactamase inhibitors	Amoxicillin-clavulanic acid	AMC	62.3 (172)	62.0 (101)	62.8 (71)
3rd generation cephalosporins	Cefatriaxone	AXO	37.3 (103)	45.4 (74)	25.7 (29)
Folate pathway inhibitors	Trimethoprim-sulfamethoxazole	SXT	76.1 (210)	82.8 (135)	66.4 (75)
1st generation Quinolones	Nalidixic acid	NAL	60.5 (167)	63.8 (104)	55.8 (63)
3rd generation Quinolones	Ciprofloxacin	CIP	33.0 (91)	39.9 (65)	23.0 (26)
Penicillins	Temocillin	TEM	12.3 (34)	17.8 (29)	4.4 (5)
Polymyxins	Colistin	CST	6.9 (19)	10.4 (17)	1.8 (2)
Carbapenems	Imipenem	IMI	15.6 (43)	22.1 (36)	6.2 (7)

Table S4. Percentage of ST and ST clonal complex of the pet-derived *E. coli* in 2018.

ST	ST clonal complex (CC)	No. of isolates	Percentage (%）	No. of isolates of ST clonal complex	Percentage of ST clonal complex (%）
12	ST12 CC	18	6.52%	23	8.33%
961	ST12 CC	5	1.81%		
10	ST10 CC	8	2.90%	22	7.97%
48	ST10 CC	6	2.17%		
167	ST10 CC	2	0.72%		
209	ST10 CC	1	0.36%		
617	ST10 CC	4	1.45%		
1638	ST10 CC	1	0.36%		
73	ST73 CC	10	3.62%	17	6.16%
1262	ST73 CC	6	2.17%		
1735	ST73 CC	1	0.36%		
101	ST101 CC	13	4.71%	17	6.16%
359	ST101 CC	4	1.45%		
648	ST648 CC	11	3.99%	12	4.35%
2011	ST648 CC	1	0.36%		
23	ST23 CC	2	0.72%	11	3.99%
88	ST23 CC	4	1.45%		
90	ST23 CC	1	0.36%		
410	ST23 CC	2	0.72%		
423	ST23 CC	1	0.36%		
2188	ST23 CC	1	0.36%		
58	ST155 CC	7	2.54%	10	3.62%
155	ST155 CC	3	1.09%		
448	ST448 CC	7	2.54%	7	2.54%
156	ST156 CC	5	1.81%	5	1.81%
453	ST86 CC	2	0.72%	3	1.09%
641	ST86 CC	1	0.36%		
46	ST46 CC	3	1.09%	3	1.09%
162	ST469 CC	3	1.09%	3	1.09%
206	ST206 CC	2	0.72%	3	1.09%
5086	ST206 CC	1	0.36%		
328	ST278 CC	3	1.09%	3	1.09%
181	ST168 CC	2	0.72%	2	0.72%
38	ST38 CC	2	0.72%	2	0.72%
398	ST398 CC	2	0.72%	2	0.72%
1737	ST205 CC	1	0.36%	1	0.36%
349	ST349 CC	1	0.36%	1	0.36%

602	ST446 CC	1	0.36%	1	0.36%
13	ST13 CC	1	0.36%	1	0.36%
131	ST131 CC	1	0.36%	1	0.36%
69	ST69 CC	1	0.36%	1	0.36%
2165	None	8	2.90%	/	/
127	None	6	2.17%	/	/
3285	None	6	2.17%	/	/
75	None	5	1.81%	/	/
224	None	5	1.81%	/	/
372	None	5	1.81%	/	/
929	None	5	1.81%	/	/
117	None	4	1.45%	/	/
457	None	4	1.45%	/	/
2086	None	4	1.45%	/	/
2569	None	4	1.45%	/	/
83	None	3	1.09%	/	/
211	None	3	1.09%	/	/
4212	None	3	1.09%	/	/
6015	None	3	1.09%	/	/
6422	None	3	1.09%	/	/
6560	None	3	1.09%	/	/
7865	None	3	1.09%	/	/
8682	None	3	1.09%	/	/
409	None	2	0.72%	/	/
942	None	2	0.72%	/	/
1147	None	2	0.72%	/	/
1196	None	2	0.72%	/	/
1290	None	2	0.72%	/	/
1716	None	2	0.72%	/	/
2175	None	2	0.72%	/	/
2522	None	2	0.72%	/	/
2690	None	2	0.72%	/	/
187	None	1	0.36%	/	/
327	None	1	0.36%	/	/
603	None	1	0.36%	/	/
697	None	1	0.36%	/	/
744	None	1	0.36%	/	/
761	None	1	0.36%	/	/
971	None	1	0.36%	/	/
1118	None	1	0.36%	/	/
1495	None	1	0.36%	/	/
2164	None	1	0.36%	/	/
2178	None	1	0.36%	/	/

2448	None	1	0.36%	/	/
4946	None	1	0.36%	/	/
4995	None	1	0.36%	/	/
5131	None	1	0.36%	/	/
5683	None	1	0.36%	/	/
7063	None	1	0.36%	/	/

“/” indicates isolates that are not belonging to any ST CC.

Table S5 Basic information of pets.

Category	Sub-category	Number of animals	Percentage （%）
Age	0~1Y	62	45.26
	1~6Y	56	40.88
	6Y~	19	13.87
Sex	Male	79	57.66
	Female	58	42.34
Breed(cat)	British Shorthair cat	20	37.74
	Local species	12	22.64
	Dragon Li	5	9.43
	Ragdoll	5	9.43
	Chausie	1	1.89
	Siamese	2	3.77
	Garfield	2	3.77
	American Shorthair	2	3.77
	Scottish Fold	1	1.89
	Chinchilla	1	1.89
	Maine	1	1.89
	British shorthair cat (blue fur)	1	1.89
Breed(dog)	Poodle	19	22.62
	Welsh corgi pembroke	13	15.48
	Local species	8	9.52
	Siberian husky	6	7.14
	Border collie	4	4.76
	Labrador retriever	5	5.95
	Bichon Frise	6	7.14
	Shiba Inu	4	4.76
	West highland white terrier	2	2.38
	French bulldog	2	2.38
	Beagle	2	2.38
	Siberian Husky	2	2.38
	Pomeranian	2	2.38
	Bedlington terriers	1	1.19
	Standard Schnauzer	1	1.19
	Canis lupus familiaris	1	1.19
	Samoyed	1	1.19
	Cocker Spaniel	1	1.19
	Canis lupus familiaris	1	1.19
	Others	3	3.57

Dataset 1. Genetic characterization of carbapenem-resistant *E. coli* from 2018.
